# Efficacy of xeno-free induced mesenchymal stem/stromal cells in enhancing autologous fat graft survival

**DOI:** 10.1007/s12282-026-01838-3

**Published:** 2026-02-23

**Authors:** Hideharu Nakamura, Takaya Makiguchi, Takahiro Yamaguchi, Sasagu Kurozumi, Daisuke Kamiya, Makoto Ikeya, Takaaki Fujii, Akihiko Uchiyama, Sei-ichiro Motegi, Jun Horiguchi, Ken Shirabe, Satoshi Yokoo

**Affiliations:** 1 Department of Oral and Maxillofacial Surgery, and Plastic Surgery, Gunma University Graduate School of Medicine, 3-39-22, Showa-cho, Maebashi, Gunma 371-8511 Japan; 2 Department of Breast Surgery, International University of Health and Welfare, Chiba, Japan; 3 Center for iPS Cell Research and Application (CiRA), Kyoto University, Kyoto, Japan; 4 Department of Breast Surgery and Oncology, Nippon Medical School Hospital, Tokyo, Japan; 5 Department of Dermatology, Gunma University Graduate School of Medicine, Gunma, Japan; 6 Department of General Surgical Science, Gunma University Graduate School of Medicine, Gunma, Japan

**Keywords:** Mesenchymal stem cells, Induced pluripotent stem cells, Xeno-free induced mesenchymal stem/stromal cells, Fat grafting, Breast reconstruction

## Abstract

**Background:**

Breast reconstruction using autologous fat grafting offers a less invasive alternative with shorter surgical times compared to traditional flap procedures. Although adipose-derived stem cells (ASCs) have improved fat graft survival rates, the variability in mesenchymal stem cells (MSCs) quality due to donor health and medication intake poses challenges. MSCs derived from induced pluripotent stem cells (iPSC-MSCs) demonstrate enhanced proliferative and immunomodulatory properties that may further enhance outcomes.

**Methods:**

We harvested human adipose tissue from surgical specimens and isolated ASCs. We used human iPSC-MSCs generated through a neural crest cell lineage under xeno-free conditions (iMSCs), to assess the improvement in survival and integration of autologous human adipose tissue grafts in BALB/c-nu/ + mice. We compared outcomes in groups treated with phosphate-buffered saline (PBS), ASCs, and iMSCs over 28 days, focusing on fat graft retention, fibrosis, and neovascularization, and adipogenic differentiation using vimentin/perilipin immunofluorescence staining.

**Results:**

iMSC treatment significantly preserved graft area (82.3 ± 20.5%) compared to PBS controls (50.6 ± 28.7%) and ASC treatments (65.3 ± 24.7%). It also resulted in the lowest fibrosis (7,748.7 ± 6,018.6 µm^2^) and highest neovascularization (68,681.5 ± 21,363.4 µm^2^), indicating improved tissue integration and vascularization. The proportion of vimentin-positive/perilipin-positive cells—indicative of adipogenic differentiation—was significantly higher in the iMSC group (7.46%) compared to PBS (4.51%, p = 0.025).

**Conclusion:**

iMSCs may enhance fat graft survival through both paracrine signaling and adipogenic differentiation, supporting a dual mechanism of action. These findings highlight their potential as a standardized, high-performance cellular adjunct for improving surgical outcomes in breast reconstruction and potentially other regenerative medicine applications. Further studies incorporating cell tracking techniques are warranted to confirm the direct differentiation potential of iMSCs.

## Introduction

Breast reconstruction post-mastectomy plays a pivotal role in restoring self-esteem and enhancing social engagement, facilitating smoother reintegration of breast cancer survivors into society. Traditional methods, such as autologous tissue reconstruction using flaps, provide permanent solutions, but involve lengthy surgical times, significant invasiveness, and potential morbidity at donor sites. In contrast, breast reconstruction using autologous fat grafting, though less invasive and requiring shorter surgical times than flap procedures, suffers from low fat graft survival rates of about 50% [1], with problems such as reabsorption of transplanted fat, central necrosis, and formation of oil cysts and calcification [2].

Adipose-derived stem cells (ASCs) are used to augment fat grafting and improve survival rates of fat grafts [3–6], and are increasingly applied in clinical settings for breast reconstruction. However, the quality of mesenchymal stem cells (MSCs) can vary based on the donor's health status and medication intake [7]. Additionally, the number of MSCs obtainable from elderly individuals typically decreases as in vivo MSC production declines with age [8], which restricts their efficacy in larger reconstructions and in elderly or medically compromised patients.

MSCs derived from induced pluripotent stem cells (iPSC-MSCs) represent a revolutionary advance in this field. iPSCs, which can be reprogrammed from somatic cells, possess the capacity to differentiate into various tissue types and proliferate indefinitely, and iPSC-MSCs show improved proliferation, immunomodulation, and secretion of paracrine factors compared to adult MSCs [9], iPSC-MSCs can be produced in sufficient quantities, irrespective of donor variability, providing a uniform quality that is crucial for clinical applications. Addition of iPSC-MSCs to autologous fat is expected to stabilize and enhance survival rates of fat grafts, but reports on iPSC-MSCs-augmented fat grafting are still scarce internationally.

In this study, we used MSCs induced from iPSCs through neural crest cell lineage under xeno-free conditions (iMSCs) [10] to explore the potential of iMSC-augmented fat grafting through a detailed examination of adipose tissue retention, neovascularization, and fibrosis in an animal model. The results indicate that this approach may be a useful option for breast reconstruction that may aid with smooth social reintegration of breast cancer survivors.

## Materials and methods

### Animals

The use of human adipose tissue was approved by the Ethics Committee of Gunma University (Approval ID: HS2021-150), and written informed consent was obtained from all donors prior to tissue collection. All animal experimental protocols were approved by the Gunma University Committee on Animal Care and Experimentation (Approval ID: 21–028) and were conducted in accordance with established institutional guidelines and the National Institutes of Health Guide for the Care and Use of Laboratory Animals [11]. BALB/c-nu/ + mice, sourced from the Shizuoka Laboratory Animals Center, Inc., Shizuoka, Japan, were selected for the study. Experiments were conducted exclusively on six-week-old female mice to maintain consistency across trials. The mice were housed at the Gunma University Institute of Experimental Animal Research, which maintains a specific pathogen-free environment to minimize external variables.

### Adipose-derived stem cells

Human ASCs were isolated from excess subcutaneous adipose tissue obtained during breast reconstruction with deep inferior epigastric perforator (DIEP) flaps at the Department of Plastic and Reconstructive Surgery, Gunma University. The donors were breast cancer patients (n = 6; age range, 47–59 years); three had received systemic chemotherapy, three had received endocrine therapy, and none had received radiotherapy prior to tissue harvest. The ASC isolation protocol followed that described by Zuk et al. and Kakudo et al. [12–14]. Briefly, the excised adipose tissue was thoroughly washed with phosphate-buffered saline (PBS) and finely minced using sterile scissors. This tissue was then enzymatically digested with 1 mg/ml collagenase type II (Sigma-Aldrich C6885, St. Louis, MO, USA) in PBS, mixed in equal parts, and incubated with agitation for 40 min at 40 °C in a 50 cm^3^ centrifuge tube. The enzymatic reaction was quenched by adding 10 ml of Dulbecco’s Modified Eagle Medium (DMEM) supplemented with 10% fetal bovine serum (FBS). Following centrifugation at 1600 rpm for 3 min, the floating adipose fraction and supernatant were discarded. The resultant cell suspension was then filtered through a 100-µm cell strainer (Corning, NY, USA) to remove debris and centrifuged again under the same conditions. The supernatant was aspirated, the pellet resuspended in an adequate volume of DMEM with 10% FBS, and the cell concentration determined. Cells were cultured in a humidified incubator at 37 °C with a 5% CO_2_ atmosphere. ASCs at passages three to five were utilized for the experiments to ensure optimal cell vigor and uniformity.

### Induced mesenchymal stem/stromal cells

iMSCs were provided by the Center for iPS Cell Research and Application, Kyoto University. Upon receipt, the frozen iMSCs were promptly thawed and subsequently cultured to ensure their viability and functionality. Cultivation of these cells was performed using PRIME-XV MSC XSFM MDF1 medium (FUJIFILM Irvine Scientific, Santa Ana, CA, USA), a specialized formulation designed to support the growth and maintenance of MSCs under xeno-free conditions. The cultured cells were maintained in a humidified incubator at 37 °C with a 5% CO_2_ atmosphere. For the experiments, only cells that had undergone three to five passages were selected to ensure consistency in cell quality and to minimize variations that could affect the reproducibility of the results.

### Flow cytometric analysis

Flow cytometry was employed to characterize the phenotypic profiles of isolated ASCs and iMSCs. A minimum of 1 × 10⁶ cells were suspended in 1.0 mL of DMEM supplemented with 1% FBS. The cell suspension was incubated with fluorochrome-conjugated monoclonal antibodies specific for CD90-PE, CD105-PE, and CD34-FITC (Beckman Coulter, USA). After staining, the labeled cells were analyzed using a Navios EX flow cytometer (Beckman Coulter, USA). After staining, the labeled cells were analyzed using a Navios EX flow cytometer (Beckman Coulter, USA). This analysis enabled the confirmation of mesenchymal stem cell marker expression and the exclusion of hematopoietic lineage contamination.

Flow cytometry was employed to characterize the phenotypic profiles of isolated ASCs and iMSCs. A minimum of 1 × 10⁶ cells were suspended in 1.0 mL of DMEM supplemented with 1% FBS. The cell suspension was incubated with fluorochrome-conjugated monoclonal antibodies specific for CD105-PE, CD90-PE, CD73-PE, and CD34-FITC (Beckman Coulter, USA). After staining, the labeled cells were analyzed using a Navios EX flow cytometer (Beckman Coulter, USA). This analysis enabled the confirmation of mesenchymal stem cell marker expression and the exclusion of hematopoietic lineage contamination.

### Animal model

The fat grafting procedure used in this study was based on the Coleman technique [15], a recognized method in the field. For the purpose of the study, 0.3 ml of human adipose tissue was harvested using the liquid overflow method from surgically obtained excess fat. The harvested adipose tissue was meticulously minced using sterile scissors and subsequently introduced into a 2.5-ml syringe and combined with a specific cell solution prepared for each experimental group: 150 µl of PBS (control group, Group A); 150 µl of PBS containing 1 × 10^5^ ASCs (Group B); and 150 µl of PBS containing 1 × 10^5^ iMSCs (Group C). A total of sixteen cell solution preparations were created for each group to ensure consistency across experiments. Adipose tissue transplantation involved subcutaneous injection of the adipose tissue-cell solution mixture into two distinct sites on the dorsal surface of the animal model, utilizing an 18G needle to facilitate the procedure. Following transplantation, the grafted fat sites were monitored over a period of 28 days to assess the viability and integration of the grafts within the host tissue. Comprehensive analysis was performed on the extracted adipose tissue at the end of the observation period, on the 28th day, to evaluate the outcomes of the fat grafting procedure and the effects of the administered cell solutions.

### Measurement of adipose tissue size

The dimensions of the transplanted adipose tissue were quantified in square millimeters (mm^2^) with the width-by-length method using calipers, as previously described [16]. To monitor the progression of tissue integration and growth, measurements were systematically taken at four-day intervals. This assessment regimen resulted in a total of eight measurements spanning the duration of the study, culminating on the 28th day post-transplantation.

### Morphological assessment of adipose tissue

To evaluate the morphological characteristics of adipocytes within the three types of transplanted adipose tissue, samples were initially fixed in 10% formalin and subsequently embedded in paraffin. Thin sections of the embedded tissues were prepared and subjected to hematoxylin and eosin (H&E) staining to highlight cellular and tissue structures. These sections were then examined using light microscopy to assess adipocyte morphology. For a comprehensive analysis, sixteen fields at 100 × magnification were randomly selected across four H&E-stained slides for each adipose tissue type. Within these fields, the sizes of ten adjacent adipocytes were measured to determine the average adipocyte size in square micrometers (µm^2^). This process involved digital photography of each selected field, followed by precise measurement of adipocyte sizes on the photographs using ImageJ software (v.1.54, National Institutes of Health, Bethesda, MD), in alignment with protocols outlined in prior research [17]. This methodology enabled quantitative assessment of adipocyte size variations among the different transplant types, facilitating a detailed understanding of their morphological characteristics.

### Assessment of fibrosis in transplanted adipose tissue

To quantify the extent of fibrosis within the transplanted adipose tissues, sections were subjected to Azan Mallory staining. Following fixation in 10% formalin and paraffin embedding, tissue sections were cut to a thickness of 4 µm. These sections then underwent a comprehensive deparaffinization process before being subjected to a series of staining steps designed to accentuate fibrotic regions. Specifically, the protocol involved incubating the specimens in Azocarmine G-drifting solution for 60 min, followed by treatment with 5% aqueous phosphorus molybdate. Subsequently, the sections were exposed to 30% hydrogen peroxide for 15 min and then stained with a mixture of Aniline blue and Orange G for 10 min. The staining process was completed using a series of dehydration, immersion, and occlusion steps to prepare the specimens for microscopic evaluation. For quantitative assessment of fibrosis, sixteen fields at 40 × magnification were randomly selected from four Azan Mallory-stained slides per adipose tissue type. The fibrotic area within each field was measured in square micrometers (µm^2^) using ImageJ software [18].

### Assessment of neovascularization in adipose tissue

To evaluate neovascularization within the transplanted adipose tissues, capillary structures were identified through immunohistochemical staining using the von Willebrand factor, a marker that is widely used for highlighting endothelial cells in neovascular networks. The staining protocol started with an overnight incubation of adipose tissue sections at 4 °C with a rabbit polyclonal anti-von Willebrand Factor antibody (Abcam plc., Cambridge, UK). This initial step was followed by application of a biotinylated secondary antibody and then an HRP-conjugated anti-rabbit IgG goat polyclonal antibody (Nichirei Bioscience, Tokyo, Japan) to amplify the signal for antigenic detection. Visualization of antigen–antibody complexes was achieved using 3,3'-diaminobenzidine (DAB) as a chromogen, and the sections were subsequently counterstained with hematoxylin to enhance cellular detail. For quantitative analysis of neovascularization, sixteen fields at 40 × magnification were randomly selected from four immunostained slides corresponding to each type of transplanted adipose tissue. The area covered by neovascularization in each field was measured in square micrometers (µm^2^) using ImageJ software.

The fields for analysis were selected specifically from the graft margin—the transitional zone between the transplanted adipose tissue and host subcutaneous tissue—where angiogenic activity is typically highest. This region was defined histologically by the interface showing infiltration of host cells and new capillary structures, and was chosen to evaluate the contribution of MSCs to vascular ingrowth at the graft-host interface.

### Histological analysis

Immunofluorescence staining was conducted to evaluate the presence and differentiation of transplanted stem cells within the adipose tissue. Paraffin-embedded sections were deparaffinized, rehydrated, and subjected to antigen retrieval by microwaving in 10 mM citrate buffer (pH 6.0). After blocking with 3% bovine serum albumin (BSA), sections were incubated overnight at 4 °C with the following primary antibodies: anti‑vimentin antibody (clone V9, catalog no. VIM‑V9‑L, Leica Biosystems, Germany), which recognizes human-specific vimentin, and anti‑perilipin A antibody (clone DBM15.6, catalog no. MOB534 [concentrated] or PDM534 [prediluted], Diagnostic BioSystems, USA), a marker of mature adipocytes. Fluorescently labeled secondary antibodies (Opal520 and Opal570; Akoya Biosciences, USA) were applied, followed by nuclear counterstaining with DAPI. Vimentin served as a marker for human mesenchymal stem cells (green), while perilipin indicated mature adipocytes (red). Quantitative analysis was performed using ImageJ software. The number of cells positive for both vimentin and perilipin (double-positive cells) was expressed as a percentage of the total number of perilipin-positive cells in each image field. Prior to detailed histological evaluation, low-magnification images were examined to identify structural zones within the grafted fat: peripheral capsule, viable fat tissue, and necrotic or fibrotic core. All subsequent analyses were limited to regions rich in viable adipocytes, carefully avoiding areas with apparent necrosis or dense fibrosis. Region-of-interest (ROI) selection was standardized across samples to minimize observer bias.

### Statistical analysis

Statistical significance was evaluated using one-way analysis of variance (ANOVA), followed by a Tukey post hoc test, as deemed appropriate for comparison of groups. Prior to performing one-way ANOVA, data were tested for normality using the Shapiro–Wilk test and for homogeneity of variances using Levene’s test. Standard errors of the mean are depicted by error bars in graphical representations, with the number of experiments (n) stated for each experimental condition. Data were analyzed using EZR (Saitama Medical Center, Jichi Medical University, Saitama, Japan), a graphical user interface designed for R (The R Foundation for Statistical Computing, Vienna, Austria, v.4.1.2). EZR extends the capabilities of R Commander (v.2.7–1) by incorporating additional statistical functions that are commonly used in biostatistical analysis [19].

## Results

### Characterization of ASCs and iMSCs

**Fig. 1:**
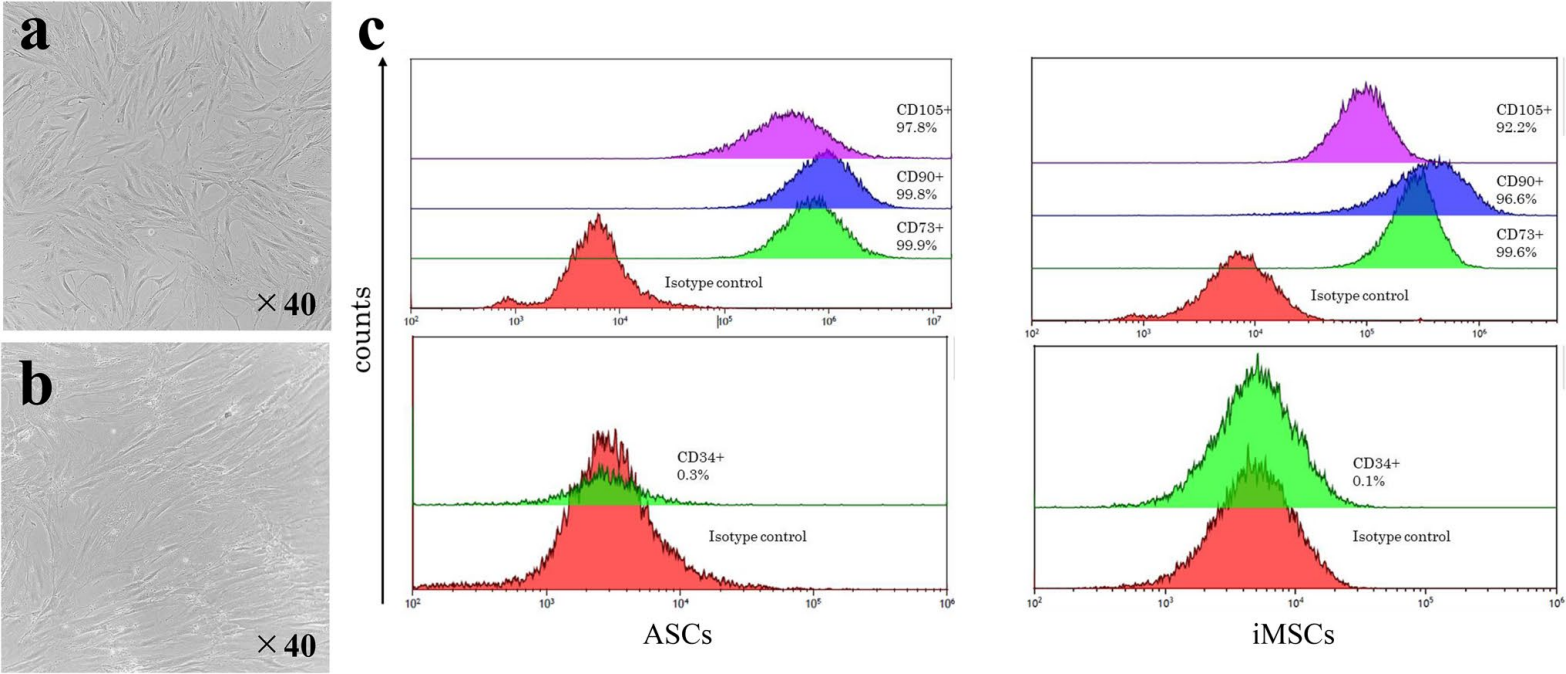
Characterization of ASCs and iMSCs. **a** Phase-contrast microscopic image of adipose-derived stem cells (ASCs). **b** Phase-contrast microscopic image of induced mesenchymal stem/stromal cells (iMSCs). **c** Flow cytometric analysis showing high expression of mesenchymal stem cell markers CD105, CD90 and CD73 in both ASCs and iMSCs, with minimal expression of the hematopoietic marker CD34

The morphology of mesenchymal stem cells isolated from human adipose tissue and iMSCs was observed under optical microscopy and exhibited characteristics typical of MSCs. Flow cytometric analysis confirmed high expression levels of positive markers (CD105, CD90, and CD73) and minimal expression of the negative marker CD34. Specifically, ASCs showed 97.8%, 99.8%, and 99.9% positivity for CD105, CD90, and CD73, respectively, compared to 0.3% for CD34. iMSCs exhibited a similar profile, with 92.2%, 96.6%, and 99.6% positivity for the respective markers, and only 0.1% for CD34 (Fig. 1). These findings confirmed that both ASCs and iMSCs possess the characteristic immunophenotype of mesenchymal stem cells.

### Adipose tissue size

**Fig. 2:**
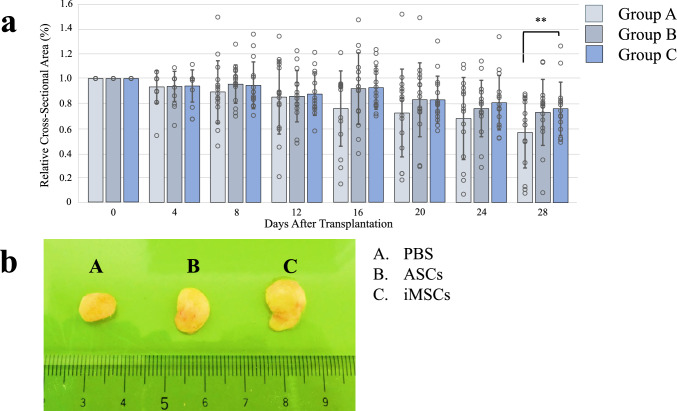
Quantitative analysis of transplanted fat grafts. **a** Quantification of graft area. Adipose tissue (0.3 ml) was transplanted subcutaneously with 150 µl PBS (Group A); 150 µl PBS containing 1 × 10^5^ ASCs (Group B); and 150 µl PBS containing 1 × 10^5^ iMSCs (Group C). Graft area was measured from histological sections and expressed as a percentage relative to the baseline (Day 0) cross-sectional area. These values reflect two-dimensional area only, and do not represent actual volume or weight. By day 28, Group C showed significantly greater retained area compared to Group A (P < 0.01; n = 16 per group). Data are shown as mean ± standard error of the mean (SEM). **b** Representative images of the extirpated transplanted fat on day 28 post-transplantation

Adipose tissue (0.3 mL) was combined with 150 µl of PBS (control group, Group A), 150 µl PBS containing 1 × 10^5^ASCs (Group B), and 150 µl PBS containing 1 × 10^5^ iMSCs (Group C). These mixtures were subcutaneously transplanted into the dorsal region of immunodeficient mice. Throughout the 28-day post-transplantation observation period, there were no mortalities among the animals. The dimensional stability of the transplanted adipose tissues was monitored at four-day intervals. Graft size was quantified as the relative cross-sectional area by comparing the measured area at each time point with that at the time of transplantation. By day 28, the relative graft area in Groups A, B, and C was 56.8 ± 28.8%, 73.1 ± 26.7%, and 76.0 ± 21.6%, respectively. Group C exhibited a significantly larger preserved graft area compared with Group A (p = 0.003) (Fig. 2). No statistically significant difference was observed between Group B and Group C.

### Morphological evaluation of adipose tissue

**Fig. 3:**
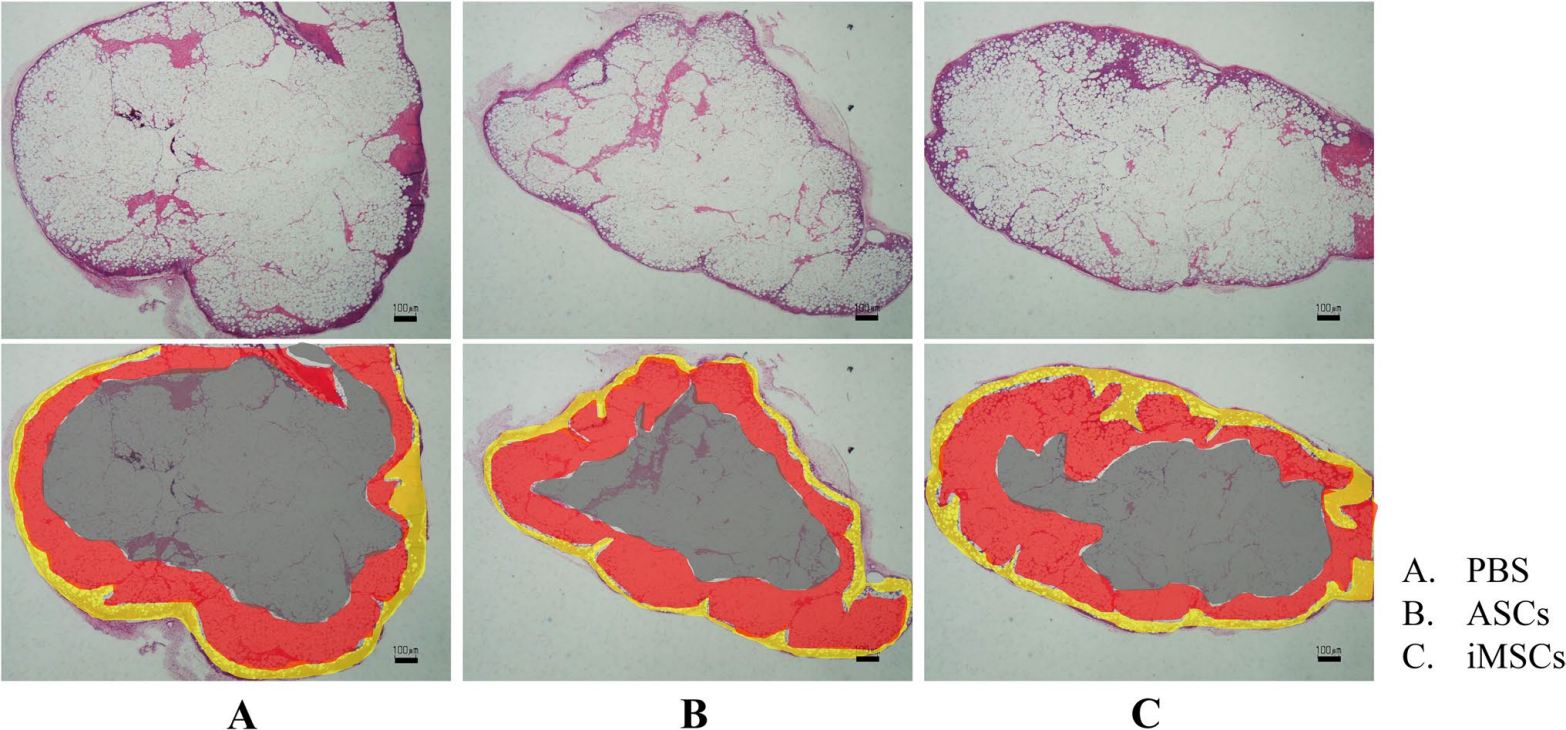
Low-magnification overview and structural characteristics of transplanted adipose tissue. A representative overview of grafted adipose tissue shows three distinct zones: the outer capsule region (yellow), the area containing viable adipocytes (red), and the necrotic or fibrotic central area (black). Morphological (Fig. 4), fibrotic (Fig. 5), neovascularization (Fig. 6), and immunofluorescence (Fig. 7) evaluations were all performed specifically within the viable adipocyte zone (red) to ensure consistency and exclude artifact-prone necrotic regions

**Fig. 4:**
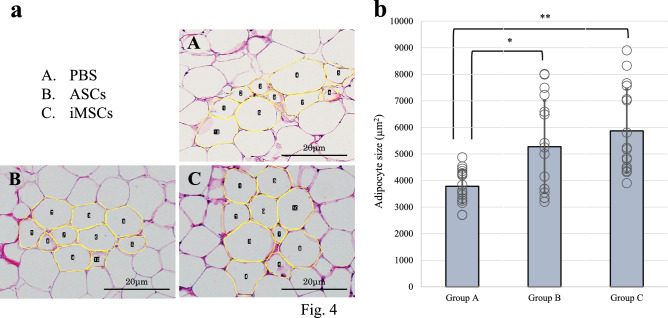
Morphological evaluation of adipose tissue. **a** Representative micrographs of Hematoxylin and Eosin (H&E)-stained adipose tissue sections from Group A (PBS), Group B (ASCs), and Group C (iMSCs). Adipocytes are outlined in yellow to illustrate differences in size among groups. Scale bars = 20 μm. **b** Quantitative analysis of adipocyte size (µm^2^). Adipocyte were significantly larger in Groups B and C compared to Group A, indicating enhanced cellular growth in the ASC- and iMSC-treated groups. Data are shown as mean ± standard error of the mean (SEM). *P < 0.05, **P < 0.01 (n = 16 per group)

On the 28th day post-transplantation, adipose tissues were extracted and processed for histological examination. Tissues were stained with H&E and analyzed under a light microscope to assess morphological features. To clarify the histological evaluation context, we first assessed the structural features of the grafted adipose tissue using low-magnification overview images. These revealed three concentric zones: (1) an outer capsule-like boundary, (2) a viable adipocyte-rich area, and (3) a central necrotic or fibrotic region. All quantitative analyses of adipocyte morphology, fibrosis, neovascularization, and immunofluorescence staining were consistently performed in the viable adipocyte zone to avoid regional bias and ensure comparability across groups (Fig. 3). The mean adipocyte sizes for Groups A, B, and C were 3783.4 ± 639.2 µm^2^, 5275.3 ± 1731.3 µm^2^, and 5869.7 ± 1572.6 µm^2^, respectively. The adipose tissues in Groups B and C had significantly larger adipocytes compared to Group A (p = 0.01, p = 0.0005, respectively) (Fig. 4). No statistically significant difference in adipocyte size was observed between Group B and Group C. These findings suggest that adipose tissues augmented with ASCs or iMSCs maintained a larger area and more favorable morphology at 28 days post-transplantation, although the lack of baseline adipocyte size measurements limits interpretation regarding true tissue retention.

In H&E stained sections, grafted fat tissue exhibited typical zonal architecture, including a peripheral capsule, viable adipocyte-rich areas, and in some cases, a central zone with reduced cellular density. However, no obvious signs of necrotic adipocytes—such as disrupted cell membranes, cytoplasmic fragmentation, or ghost-like adipocyte outlines—were observed in any group. These observations suggest that the grafted tissues maintained overall viability by day 28.

### Fibrosis of adipose tissue

**Fig. 5:**
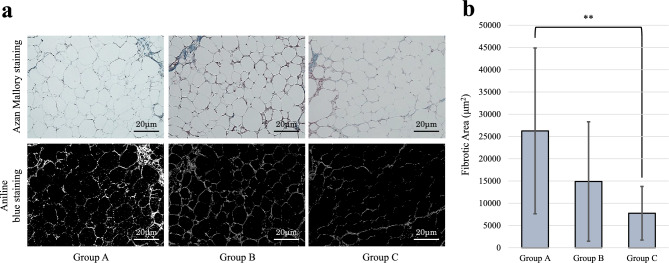
Fibrosis of transplanted adipose tissue. **a** Representative micrographs of adipose tissue sections from Group A (PBS), Group B (ASCs), and Group C (iMSCs) with an Azan Mallory stain, showing fibrosis. Lower panels display the separation of blue staining to highlight areas of fibrosis. **b** Quantitative analysis of the fibrotic area (mm^2^). Adipose tissues augmented with iMSCs (Group C) had significantly smaller fibrotic areas compared to those treated with PBS (Group A). Data are shown as mean ± standard error of the mean (SEM). *P < 0.05, **P < 0.01 (n = 16 per group). Scale bars = 20 μm

Sections of extracted adipose tissues were subjected to Azan Mallory staining for assessment of fibrosis. The fibrotic areas on day 28 post-transplantation in Groups A, B, and C were 26,247.4 ± 18,624.6 µm^2^, 14,889.1 ± 13,418.4 µm^2^, and 7,748.7 ± 6,018.6 µm^2^, respectively, and Group C had a significantly smaller area of fibrosis compared to Group A (p = 0.001) (Fig. 5). Although the fibrosis area was lowest in the Group C, there was no statistically significant difference between the Group C and Group B. These findings suggest that augmenting adipose tissue with iMSCs may suppress fibrosis and improve tissue elasticity.

### Neovascularization in transplanted adipose tissue

**Fig. 6:**
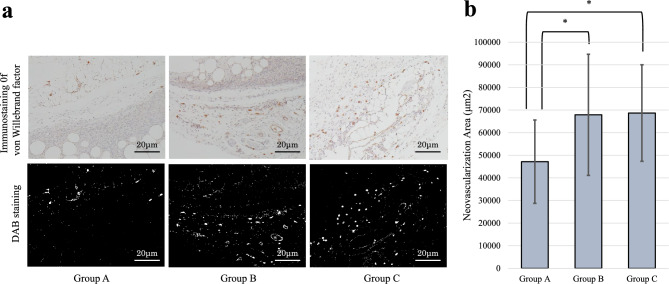
Neovascularization of transplanted adipose tissue. **a** Representative micrographs of adipose tissue sections from Group A (PBS), Group B (ASCs), and Group C (iMSCs) with von Willebrand factor immunohistochemical staining (40 × magnification). Lower panels show 3,3'-diaminobenzidine (DAB) staining to highlight areas of neovascularization. All images were taken from the graft margin—the interface between transplanted adipose tissue and host tissue—where angiogenic activity was most pronounced. Scale bars: 20 μm. **b** Quantitative analysis of the neovascularization area (mm^2^). Adipose tissues in Group B and Group C had significantly larger areas of neovascularization compared to those in Group A. Data are shown as mean ± standard error of the mean (SEM). *P < 0.05 (n = 16 per group)

Immunostaining against von Willebrand factor was conducted on extracted adipose tissue to evaluate neovascularization, with areas quantified by DAB staining. On day 28 post-transplantation, the measured neovascularization areas for Groups A, B, and C were 47,171.0 ± 18,419.3 µm^2^, 67,892.6 ± 26,782.8 µm^2^, and 68,681.5 ± 21,363.4 µm^2^, respectively. Adipose tissues in Groups B and C had significantly larger areas of neovascularization compared to Group A (p = 0.03, p = 0.02, respectively) (Fig. 6). Although Group C exhibited the highest neovascularization among the groups, the difference between Group B and Group C did not reach statistical significance.

The neovascularization observed in this study was primarily localized to the graft margin. Quantification was performed in this region to reflect active zones of host-derived vascular ingrowth, which are critical for graft survival. This margin represents the biologically relevant interface between transplanted tissue and recipient site, where angiogenesis is functionally meaningful.

These findings suggest that augmentation with either ASCs or iMSCs supports formation of a higher density of new blood vessels, which is beneficial for tissue engraftment.

### Histological analysis

**Fig. 7:**
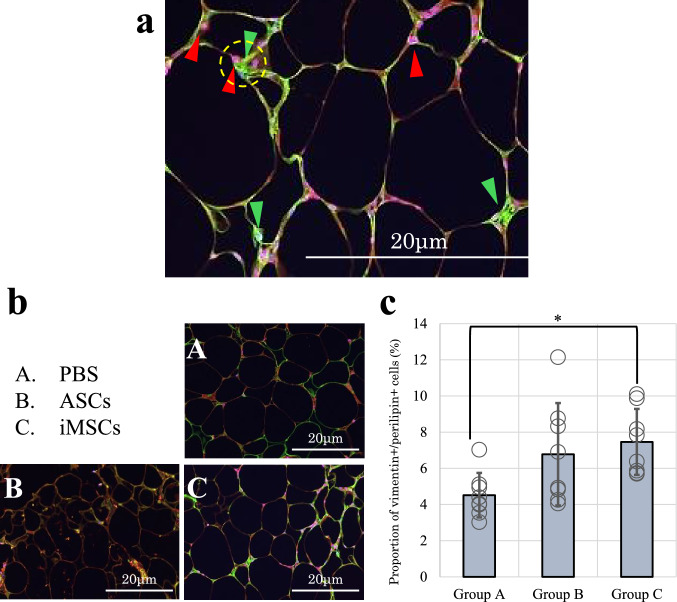
Immunofluorescence staining for verifying the survival and retention of transplanted stem cells in adipose tissue. **a** Representative high-magnification immunofluorescence images highlighting marker-positive cells. Vimentin-positive cells are indicated by green triangles, perilipin-positive adipocytes by red triangles, and cells showing co-localized expression of vimentin and perilipin (double-positive cells) are outlined by yellow dotted circles. **b** Immunofluorescence staining of adipose tissue on day 28 post-transplantation to evaluate the survival and differentiation of transplanted stem cells. Perilipin (red) marks adipocytes, and vimentin (green) marks mesenchymal stem cells. Co-localization of both markers (yellow) is more prominent in Group C (iMSCs), suggesting a higher proportion of stem cell-derived adipocytes. **c** Quantification of vimentin-positive/perilipin-positive cells expressed as a percentage of perilipin-positive cells. Group C showed a significantly higher proportion compared to Group A (*P < 0.05, n = 8 per group). Scale bar = 20 μm

Immunofluorescence staining was performed to evaluate the presence of cells co-expressing vimentin and perilipin within the transplanted adipose tissue. Vimentin-positive/perilipin-positive cells were defined as adipocytes showing co-localized expression of both markers and were quantified as a percentage of total perilipin-positive cells. The proportions of vimentin-positive/perilipin-positive cells in Groups A, B, and C were 4.51 ± 1.23%, 6.78 ± 2.83%, and 7.46 ± 1.82%, respectively. Group C exhibited a significantly higher proportion compared to Group A (p = 0.025). Although the value in Group C was also higher than in Group B, the difference was not statistically significant (p = 0.789). Representative high-magnification merged images demonstrated cells with co-localized vimentin and perilipin signals, which were predominantly observed at the graft margin (Fig. 7).

## Discussion

Recent developments in diagnostic and treatment techniques have improved the prognosis for patients with early-stage breast cancer, but this condition still requires surgery. The loss of a breast is a great emotional burden for many women, and favorable long-term prognoses for many cases of breast cancer cause this loss to have an important impact on quality of life. In breast cancer surgery, the importance of complete removal of the cancer and maintenance of cosmetic appearance has been widely debated for many years, but has yet to be fully resolved. Partial mastectomy is sometimes chosen for maintenance of cosmetic appearance, as the nipple and breast tissue distal to the lesion are preserved [20]. The defect after cancer removal is supplemented with the surrounding preserved mammary gland and fatty tissue during surgery, but the overall volume may not be sufficient to maintain a good cosmetic appearance. Thus, breast reconstruction using autologous fat grafting is used for reconstruction after partial mastectomy. In contrast to autologous tissue reconstruction using skin flaps, autologous fat grafting requires less sacrifice of the donor site, takes less time to perform, and is less invasive. However, the graft survival rate is only about 50% [1] and there is a high risk of calcification, cyst formation, and infection.

MSCs, initially isolated from bone marrow, are multipotent adult stem cells that are also found in adipose tissue, synovium, dental pulp, and umbilical cord blood. These cells can differentiate into osteocytes, chondrocytes, and adipocytes [21, 22]. MSCs play a crucial role in tissue repair and regeneration through various mechanisms, including homing to damaged tissues and modulating immune responses [23, 24]. The cells primarily exert their therapeutic effects through paracrine actions, secreting bioactive factors that stimulate resident cells to repair damaged tissue. These factors promote angiogenesis, prevent apoptosis, and enhance cell survival, proliferation, and differentiation [25].

The first surgical use of autologous fat grafting was reported by Neuber in 1893 to correct arm and orbital scars [26]. In 2001, Zuk et al. showed that adipose tissue is a source of multipotent MSCs [12]. ASCs enhance fat graft survival rates through paracrine activities and expression of growth factors that promote angiogenesis and adipogenesis, and prevent apoptosis [27, 28]. However, the quality of MSCs can vary based on the donor's health status and medication intake [7], and the number of MSCs obtainable typically decreases with age, which restricts their efficacy in larger reconstructions and in elderly or medically compromised patients [8].

iPSC-MSCs are emerging as a promising alternative to adult tissue-derived MSCs in regenerative medicine. Studies have shown that iPSC-MSCs can have comparable effects to adipose-derived MSCs in treating conditions such as inflammatory bowel disease and acute kidney injury [29, 30]. iPSC-MSCs exhibit improved proliferation, immunomodulation, and secretion of paracrine factors compared to adult MSCs [9], and maintain their differentiation potential over extended passages [31], iPSC-MSCs can be produced in sufficient quantities, regardless of donor variability, providing a uniform quality crucial for clinical applications. In the present study, we directly compared autologous fat grafting alone (Group A), ASC-assisted grafting (Group B), and iMSC-assisted grafting (Group C) using a murine model. Histological cross-sectional analysis showed that the relative cross-sectional area of grafts was significantly larger in Group C than in Group A, suggesting better maintenance of adipose tissue under iMSC treatment. However, since baseline adipocyte size and total graft volume were not recorded, the findings do not fully capture the dynamics of true volume retention. Notably, the difference in preserved graft area between Group B and Group C was not statistically significant, indicating that iMSCs did not confer a clear advantage over ASCs in this model. Similarly, while Group C exhibited larger adipocyte size and reduced fibrosis compared to Group A, these effects were not significantly different from those observed in Group B. Neovascularization was also highest in the iMSC group, but again, the difference relative to the ASC group did not reach statistical significance. Collectively, these findings suggest that iMSCs may provide supportive effects comparable to those of ASCs; however, superiority could not be demonstrated under the current experimental conditions. Furthermore, histological evaluation revealed a higher proportion of vimentin-positive/perilipin-positive cells in the iMSCs group (7.46%) compared to controls (4.51%, p = 0.025).

Vimentin is an intermediate filament protein typically expressed in mesenchymal cells and is involved in cytoskeletal organization during adipogenesis. According to the study by Heid et al., vimentin plays a pivotal structural role during early lipid droplet formation in human adipocytes, particularly through its cortical arrangement around perilipin-positive lipid inclusions [32] In our grafts, the co-expression of vimentin and perilipin indicates the presence of adipogenic lineage cells within the iMSC-treated grafts. Whether these cells are derived directly from iMSCs or indirectly from host stimulation remains to be confirmed. Future studies using cell labeling and tracking technologies will help clarify the extent of direct cellular integration versus host cell recruitment and activation.

Traditionally, the benefits of ASC-assisted fat grafting have been attributed to paracrine signaling that promotes angiogenesis and reduces inflammation [27, 28]. ASCs also contribute to adipose tissue remodeling and turnover by differentiating into adipocytes [33, 34]. Similarly, MSCs derived from iPSCs have also been reported as a promising source for adipocyte generation [35, 36], acting as progenitor cells in the maintenance and regeneration of adipose tissue. Although our findings are consistent with a potential dual mechanism—paracrine support and cellular integration—further investigation is needed to clarify the exact contribution of iMSCs to adipose tissue regeneration at the cellular level.

MSCs derived from various sources, including bone marrow, umbilical cord blood, and Wharton's jelly, can be differentiated into endothelial cells in vitro [37–40]. iPSC-derived MSCs can also be differentiated into endothelial cells [31, 41], which facilitate angiogenesis. This is critical for the viability and functionality of transplanted tissues, since it ensures that they receive a sufficient blood supply and nutrients essential for graft survival.

ASCs secrete various cytokines and growth factors, such as hepatocyte growth factor (HGF), vascular endothelial growth factor, and interleukins, which contribute to their immunomodulatory, angiogenic, and neurotrophic properties [42, 43]. HGF plays a particularly important role in enhancing the ability of ASCs to promote tissue reperfusion and inhibit fibrosis [44, 45]. Given the enhanced histological and functional outcomes observed in this study, iMSCs likely possess similar or enhanced paracrine capabilities that contribute to graft stabilization, vascularization, and adipogenesis. Collectively, these findings support the clinical potential of iMSCs as a standardized, high-performance cellular adjunct in autologous fat grafting. Future studies with longer observation periods and advanced cell tracking will further clarify the mechanisms of action and support clinical translation.

One important clinical consideration for future application of iMSCs in breast cancer patients is the potential requirement for immunosuppressive therapy, as allogeneic iMSCs may not be fully immunoprivileged. While autologous ASCs are more immunologically compatible, their availability and quality vary depending on patient age, comorbidities, and prior treatments. In the present study, ASCs were derived from breast cancer patients undergoing DIEP flap reconstruction, including donors with prior systemic therapies, reflecting a clinically relevant donor background. In contrast, iMSCs offer the advantages of consistent quality, scalable production, and xeno-free conditions suitable for Good Manufacturing Practice (GMP) environments. These features make iMSCs a viable alternative, especially in cases where ASCs are insufficient or compromised. Nevertheless, the need for immunosuppression remains a critical hurdle, and future studies should evaluate immune compatibility and tumor safety in oncologic settings before clinical application.

As a limitation of this study, graft size was assessed by two-dimensional cross-sectional area measurements (mm^2^) on histological sections. This method does not account for the total volume or weight of the grafted adipose tissue. Future studies should consider more accurate volumetric assessments, including longitudinal micro-CT imaging, water displacement techniques, or endpoint graft weight measurement. Additionally, since pre- or immediate post-grafting measurements of adipocyte size were not obtained, it remains unclear whether the observed adipocyte sizes reflect true tissue retention or compensatory hypertrophy. To account for this limitation, the present study describes graft outcomes using the term “preserved cross-sectional area” rather than “retention,” which may not fully capture the biological dynamics under the current methodology.

## Conclusion

The findings from this study underscore the potential of iMSCs to enhance preserved graft area, reduced fibrosis, and increased neovascularization in autologous fat grafts. Immunofluorescence analysis revealed a higher proportion of vimentin-positive/perilipin-positive cells in the iMSCs group, which may suggest enhanced adipogenic remodeling within the graft. However, without direct cell-labeling techniques such as GFP tracing, it remains unclear whether these cells are derived from transplanted iMSCs or represent host-derived adipocytes influenced by trophic factors. Therefore, definitive confirmation will require further investigation. In order to facilitate clinical application, comprehensive studies and clinical trials are needed to validate the safety, efficacy, and behavior of iMSCs. As our understanding of iMSCs deepens, their potential applications in breast reconstruction and other medical fields appears to be increasingly promising. This could herald a new era in regenerative medicine, potentially revolutionizing treatment paradigms across specialties by leveraging the power of advanced stem cell technologies.

## Data Availability

The datasets generated and analyzed during the current study are not publicly available due to ethical restrictions and privacy concerns related to human-derived materials, but are available from the corresponding author on reasonable request.
